# Combined association of socioeconomic status and type 2 diabetes with influenza vaccination in older adults: A cross-sectional analysis of the Korea National Health and Examination Survey (2019–2022)

**DOI:** 10.1371/journal.pone.0341831

**Published:** 2026-02-02

**Authors:** Sunghak Kim, Kyuwoong Kim

**Affiliations:** 1 Department of Media and Communication, Pusan National University, Busan, Republic of Korea; 2 National Cancer Control Institute, National Cancer Center, Goyang, Republic of Korea; 3 Graduate School of Cancer Science and Policy, National Cancer Center, Goyang, Republic of Korea; MOH Holdings Pte Ltd Singapore, SINGAPORE

## Abstract

**Background:**

Given the limited evidence on the joint association of socioeconomic status and type 2 diabetes (T2DM) with influenza vaccination uptake, we examined this association among adults aged 65 years and older eligible for the National Immunization Program in the Republic of Korea.

**Methods:**

We analyzed data from the Korea National Health and Nutrition Examination Survey (KNHANES) 2019–2022, stratified by pre-COVID-19 (2019) and COVID-19 (2020–2022) periods including 5,525 adults aged 65 years and older. The participants were classified by T2DM status and SES indicators (income, education, and economic activity). Multivariable logistic regression estimated adjusted odds ratios (aORs) and 95% confidence intervals (CIs) for influenza vaccination, adjusting for demographic characteristics, health behaviors, health status measures, and healthcare access factors.

**Results:**

Among 5,525 adults aged ≥65 years in the 2019–2022 Korea National Health and Nutrition Examination Survey, the weighted influenza vaccination rate was 77.6% overall (78.2% pre–COVID-19; 77.1% during COVID-19). Compared with high-income adults without diabetes, low-income adults with T2DM had lower odds of vaccination (adjusted odds ratio [aOR] = 0.75; 95% CI, 0.57–0.99), and low-income adults without T2DM showed a similar trend (aOR = 0.82; 95% CI, 0.65–1.03). By education, low-education adults with T2DM had lower vaccination likelihood (aOR = 0.83; 95% CI, 0.61–1.15) compared with college-educated adults without T2DM. Economic activity was not significantly associated with vaccination (aOR = 0.91; 95% CI, 0.70–1.18). Findings were consistent across pre–COVID-19 and COVID-19 periods (*P* for interaction = 0.24).

**Conclusions:**

Socioeconomic disadvantage and T2DM may jointly contributed to lower influenza-vaccination uptake among Korean adults aged 65 years and older.

## 1. Introduction

Seasonal influenza remains a major global public health concern, especially among older adults, who face a substantially higher risk of severe complications, hospitalization, and death, contributing to a considerable socioeconomic burden [1–3]. Vaccination is the most effective and cost-efficient strategy for prevention [4–7]. However, achieving equitable vaccination coverage remains challenging, with uptake varying across socioeconomic groups [8–12]. These persistent disparities suggest that universal availability of vaccination alone is insufficient to ensure equal protection among vulnerable populations, highlighting the need to identify and address underlying barriers to vaccination.

Type 2 diabetes mellitus (T2DM) adds complexity to these inequities. Individuals with T2DM have consistently exhibited lower influenza vaccination rates than those without diabetes, despite being at markedly higher risk of influenza-related hospitalization and mortality [13,14]. Among older adults, who already experience age-related physiological decline and social vulnerability, the coexistence of T2DM may intensify the consequences of under-vaccination and widen existing health disparities [15]. With the global prevalence of T2DM projected to exceed 700 million by 2045 [16], improving vaccine uptake in this group represents a pressing public health goal.

In the Republic of Korea, T2DM prevalence among adults aged 65 years and older increased to 27.6% by 2018 [17]. Although influenza-vaccination coverage in older adults reached 81.7% in 2019 [18], aggregate coverage estimates may mask substantial heterogeneity across socioeconomic status (SES) [18]. Lower SES is often associated with limited healthcare access, affordability challenges, and lower health literacy [1,18,19]. Despite extensive research examining either socioeconomic disparities or diabetes-related gaps in influenza vaccination, few population-based studies have evaluated their combined association, particularly among older adults who are eligible for universal vaccination programs. This lack of evidence limits understanding of whether socioeconomic disadvantage and T2DM interact to compound inequities in preventive care, even within settings that offer free vaccination. To address this knowledge gap, this study examined the joint association of SES and T2DM with influenza vaccination uptake among Korean adults aged 65 years and older eligible for the National Immunization Program.

## 2. Methods

### Data source and study population

**Fig 1:**
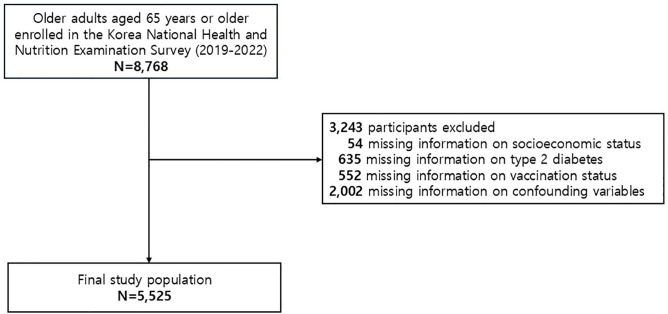
Flowchart for the study population.

Data used in this study were obtained from the Korea National Health and Nutrition Examination Survey (KNHANES), a cross-sectional survey with a stratified, multistage probability sampling design to represent the entire population of non-institutionalized civilians in the Republic of Korea. The Korea Disease Control and Prevention Agency (KCDA) collects comprehensive health and nutrition data for constructing the KNHANES through interviews, physical examinations, and laboratory tests from the selected professional investigators (i.e., nurse, nutritionist, and public health specialist) to assess the national trends of health and nutrition status. The KNHANES initially operated on a triennial schedule, commencing with the first round in 1998 and concluding with the third round in 2005. Subsequently, the survey transitioned to an annual format, implemented from the fourth round (2007–2009) and continuing to the present day. Informed consent was obtained from all participants in the KNHANES through a standardized process. Before enrollment, each participant provided written informed consent. The process ensured that participants received essential information, comprehended its content, and voluntarily agreed to participate in the study. The KNHANES has been used for a wide range of epidemiologic purpose and the detailed information is available elsewhere [20]. From the 2019–2022 Korea National Health and Nutrition Examination Survey (KNHANES VIII), a total of 8,768 adults aged 65 years or older were identified. The final analytic sample included adults aged 65 years or older with complete information on socioeconomic status, type 2 diabetes status, influenza vaccination, and prespecified confounding variables, resulting in 5,525 participants eligible for analysis (Fig 1)

### Ethics statement

All participants of the KNHANES provided written informed consent prior to the original data collection. The Institutional Review Board (IRB) of the Korea Disease Control and Prevention Agency (KCDA) approved the protocols of the research and the release of the dataset for research purposes, in adherence with the guidelines of the Declaration of Helsinki (2018-01-03-C-A).

### Definition of type 2 diabetes and socioeconomic status

Type 2 diabetes mellitus (T2DM) was defined according to the diagnostic criteria of the Korean Diabetes Association, which are consistent with those of the American Diabetes Association [22,23], as having any of the following: fasting plasma glucose ≥126 mg/dL (7.0 mmol/L), hemoglobin A1c ≥ 6.5%, or current use of antidiabetic medication. In addition, we included participants who self-reported having been diagnosed with T2DM by a physician in the KNHANES survey [21,22]. We defined socioeconomic status as a comprehensive measure of household income, education level, and economic activity to address the complexity of these determinants in social stratification and health outcomes according to previous research [23–25]. Household income was calculated by the square root adjusted mean, a method accounting for household size by dividing the total income of all members by the square root of the number of household members [26]. To facilitate cross-national comparison on household income for the period 2019–2022 in the KNHANES, household income quartile (Q). Data on education level was collected through survey interviews. Participants self-reported their highest level of education attainment from pre-defined categories including “less than elementary school”, “middle school”, “high school”, “college degree or graduate school.” Based on the distribution of collected data, education level was categorized into a binary variable, distinguishing between “less than high school” and “college or higher.” Similarly, data on economic activity were gathered via survey interviews with participants, who were asked about their current employment status. Participants indicating “currently employed” were classified as economically active, while those responding with “unemployed, economically inactive” were categorized as economically inactive.

### Assessment of confounding variables

Information on demographic, behavioral, and health-related characteristics was obtained through standardized self-reported questionnaires administered in the Korea National Health and Nutrition Examination Survey (KNHANES). Demographic variables included age (≥70 years or <70 years), sex (male or female), residential area (capital, metropolitan, or rural), type of health insurance (self-employed insured, employee insured, or medical aid), and marital status (married, bereaved, divorced, or unmarried). Health behavior variables included alcohol consumption (abstainer or never drinker vs habitual drinker, defined as consuming alcohol ≥1 time per month in the past year), cigarette smoking (non-smoker or current smoker), and physical activity (yes or no, based on performing ≥150 minutes of moderate-intensity activity, ≥ 75 minutes of vigorous-intensity activity, or an equivalent combination per week). Health status and healthcare access factors included self-perceived health status (good, normal, or poor), participation in national health screening (received or not received through the National Health Insurance Service program), comorbid conditions (hypertension, dyslipidemia, stroke, myocardial infarction, and angina pectoris), healthcare accessibility (whether needed and subsequently received medical care in the past 12 months, excluding dental services), and functional status (presence or absence of limitations in daily or social activities due to health problems). All these variables were included as covariates in the fully adjusted multivariable model (Model 3) to control for sociodemographic, behavioral, clinical, and healthcare-related confounding.

### Ascertainment of influenza vaccination status

Influenza-vaccination status was assessed through participants’ responses to the standardized questionnaire item, *“Have you been vaccinated against influenza during the past 12 months?”* Responses were recorded as a binary variable (yes/no). In the Republic of Korea, the Ministry of Health and Welfare provides influenza vaccination at no cost to adults aged 65 years or older and to infants aged 6–12 months under the National Immunization Program.

### Statistical analysis

Baseline characteristics of the study population were summarized as means with standard errors for continuous variables and as frequencies with unweighted and weighted percentages for categorical variables. Differences between participants with and without type 2 diabetes mellitus (T2DM) were evaluated using survey-weighted linear regression for continuous variables and the Rao–Scott F–adjusted chi-square test for categorical variables, accounting for the complex, stratified, multistage probability sampling design of the Korea National Health and Nutrition Examination Survey (KNHANES).

Survey weights, strata, and primary sampling units provided by KNHANES were incorporated into all analyses to generate nationally representative estimates of the Korean older-adult population. Variance estimation used Taylor-series linearization to account for the design effects of complex sampling.

To examine the combined association of socioeconomic status (SES) and T2DM with influenza vaccination, participants were classified by household income, educational attainment, and economic activity, each cross-stratified by diabetes status. Weighted multivariable logistic-regression models were used to estimate adjusted odds ratios (aORs) and 95% confidence intervals (CIs) for influenza vaccination. Model 1 included age and sex; Model 2 additionally adjusted for residential area, type of health insurance, marital status, alcohol consumption, cigarette smoking, and physical activity; and Model 3 further included self-perceived health status, participation in national health screening, comorbid conditions (hypertension, dyslipidemia, stroke, myocardial infarction, and angina pectoris), healthcare accessibility, and functional status.

Weighted prevalence estimates and absolute differences in vaccination rates were derived for each exposure group using survey procedures in SAS. Sensitivity analyses stratified by survey period (pre–COVID-19 [2019] vs COVID-19 [2020–2022]) were conducted to evaluate potential temporal interactions between SES, T2DM, and vaccination status, with P values for interaction calculated from likelihood-ratio tests.

All statistical tests were two-sided, and *P* < 0.05 was considered statistically significant. Analyses were conducted using SAS, version 9.4 (SAS Institute Inc, Cary, NC). Confidence intervals were not adjusted for multiple comparisons and should not be interpreted as formal hypothesis testing.

## 3. Results

Among 5,525 adults aged 65 years or older (weighted population ≈ 27 million), 1,617 (29.3%) had type 2 diabetes mellitus (T2DM). The demographic, socioeconomic, behavioral, and clinical characteristics of the study population are presented in Table 1. Compared with participants without T2DM, those with T2DM were older and more likely to have comorbid hypertension, dyslipidemia, myocardial infarction, and angina pectoris (all *P* < .001). Participants with T2DM were also more likely to report poor self-rated health, functional limitation, and lower healthcare accessibility (all *P* < .05). Sex distribution, alcohol consumption, smoking, physical activity, and participation in the national health-screening program were similar between groups. The overall weighted influenza-vaccination rate in the cohort was 77.6%, with slightly higher coverage in 2019 (78.2%) than during 2020–2022 (77.1%).

**Table 1:** General characteristics of adults aged 65 years or older in the Korean National Health and Nutrition Examination Survey (2019-2022) stratified by diabetes status.

Characteristics	No diabetes		Diabetes^a^		*P*-value^c^
	Unweighted	Weighted^b^	Unweighted	Weighted^b^	
**Socioeconomic status**					
**Household size, no**					
**≤ 2**	3123 (79.9)	14,190,398 (74.3)	1,290 (79.8)	5,856,290 (74.9)	0.277
**3-4**	667 (17.1)	4,135,535 (21.7)	257 (15.9)	1,559,869 (20)	
**≥5**	118 (3.0)	774,102 (4.0)	70 (4.3)	399,697 (5.1)	
**Household income**^**d**^					0.051
**1**^**st**^ **quartile**	943 (24.1)	4,297,829 (22.5)	438 (27.1)	2,040,681 (26.1)	
**2**^**nd**^ **quartile**	961 (24.6)	4,403,087 (23.1)	417 (25.8)	1,843,829 (23.6)	
**3**^**rd**^ **quartile**	1,006 (25.7)	4,927,125 (25.8)	379 (23.4)	1,845,170 (23.6)	
**4**^**th**^ **quartile**	998 (25.6)	5,471,994 (28.6)	383 (23.7)	2,086,176 (26.7)	
**Education level**					0.429
**≥ College**	529 (13.5)	2846706 (14.9)	1,420 (87.8)	6,731,618 (86.1)	
**≤ High school**	3379 (86.5)	16,253,329 (85.1)	197 (12.2)	1,084,238 (13.9)	
**Economically active**					
**Yes**	529 (13.5)	2,846,706 (14.9)	585 (36.2)	2,779,245 (35.6)	0.06
**No**	3,379 (86.5)	16,253,329 (85.1)	1,032 (63.8)	5,036,612 (64.4)	
**Demographic factors**					
**Age, years**					
**≥ 70**	1,606 (41.1)	8,163,760 (42.7)	606 (37.5)	3,030,196 (38.8)	0.016
**< 70**	2,302 (58.9)	10,936,276 (57.3)	1,011 (62.5)	4,785,661 (61.2)	
**Sex**					
**Male**	1,694 (43.3)	8,315,756 (43.5)	754 (46.6)	3,633,977 (46.5)	0.091
**Female**	2,214 (56.7)	10,784,279 (56.5)	863 (53.4)	4,181,879 (53.5)	
**Residential area**					
**Capital**	683 (17.5)	3,967,345 (20.8)	233 (14.4)	1,416,403 (18.1)	0.098
**Metropolitan**	1750 (44.8)	8,720,211 (45.7)	762 (47.1)	3,781,846 (48.4)	
**Rural (city/town)**	1475 (37.7)	6,412,480 (33.6)	622 (38.5)	2,617,607 (33.5)	
**Type of health insurance**					
**Self-employed insured**	1308 (33.5)	6,420,642 (33.6)	553 (34.2)	2,702,348 (34.6)	0.001
**Employee insured**	2355 (60.3)	11,608,782 (60.8)	917 (56.7)	4,460,331 (57.1)	
**Medical aid**	245 (6.2)	1,070,611 (5.6)	147 (9.1)	653,177 (8.4)	
**Marital status**					
**Married (living together)**	2738 (70.1)	13,368,905 (70)	1,077 (66.6)	5,189,565 (66.4)	0.192
**Married (living separately)**	42 (1.1)	209,305 (1.1)	15 (0.9)	78,067 (1.0)	
**Bereaved**	926 (23.7)	4,522,680 (23.7)	418 (25.9)	2,062,205 (26.4)	
**Divorced**	171 (4.4)	842,620 (4.4)	90 (5.6)	417,961 (5.3)	
**Unmarried**	31 (0.8)	156,526 (0.8)	17 (1.1)	68,058 (0.9)	
**Health Behavior**					
**Alcohol consumption**					0.327
**Abstainer/Never drinker**^**e**^	2,570 (65.8)	1,2547,166 (65.7)	1,090 (67.4)	5,259,267 (67.3)	
**Habitual consumption**^**f**^	1,338 (34.2)	6,552,870 (34.3)	527 (32.6)	2,556,589 (32.7)	
**Cigarette smoking**					0.274
**Non-smoker**	3,557 (91.0)	17,327,011 (90.7)	1,454 (89.9)	7,001,601 (89.6)	
**Current smoker**	351 (9)	1,773,024 (9.3)	163 (10.1)	814,256 (10.4)	
**Physical activity** ^**g**^					0.166
**Yes**	2,662 (68.1)	12,812,920 (67.1)	1,121 (69.3)	5,413,311 (69.3)	
**No**	1,246 (31.9)	6,287,116 (32.9)	496 (30.7)	2,402,545 (30.7)	
**Self-perceived health status**					
**Good**	1,049 (26.8)	5,314,410 (27.8)	319 (19.7)	1,601,282 (20.5)	<0.001
**Normal**	1,867 (47.8)	9,097,114 (47.6)	782 (48.4)	3,762,463 (48.1)	
**Bad**	992 (25.4)	4,688,511 (24.5)	516 (31.9)	2,452,112 (31.4)	
**Comorbid conditions**					
**Hypertension**	2,641 (67.6)	12,504,723 (65.5)	1,075 (66.5)	5,305,366 (67.9)	<0.001
**Dyslipidemia**	1,233 (31.6)	6,129,387 (32.1)	761 (47.1)	3,762,399 (48.1)	<0.001
**Stroke**	117 (3.0)	554,952 (2.9)	71 (4.4)	303,917 (3.9)	0.103
**Myocardial infarction**	67 (1.7)	329,394 (1.7)	54 (3.3)	259,314 (3.3)	0.001
**Angina pectoris**	122 (3.1)	561,502 (2.9)	97 (6.0)	440,642 (5.6)	<0.001
**Limited functional status** ^**h**^	378 (9.7)	1,665,097 (8.7)	190 (11.8)	842,949 (10.8)	0.023
**Healthcare accessibility** ^**i**^	2,641 (67.6)	12,504,723 (65.5)	1,156 (71.5)	5,376,970 (68.8)	0.026
**Health Screening** ^**j**^					
**Received**	2,945 (75.4)	1,4342,914 (75.1)	1,179 (72.9)	572,3313 (73.2)	0.223
**Not received**	963 (24.6)	4,757,121 (24.9)	438 (27.1)	2,092,544 (26.8)	
**Fasting serum glucose, mg/dL (SE)**	98.3 (0.1)	98.4 (0.2)	127.8 (0.8)	127.5 (0.8)	<0.001
**HbA1c, % (SE)**	5.7 (0.0)	5.7 (0.0)	7.0 (0.0)	7.0 (0.0)	<0.001

^a^Defined as fasting serum glucose ≥126 mg/dL or use of antidiabetic medication or history of type 2 diabetes diagnosis by physicians or glycated hemoglobin ≥6.5%.

^b^Sampling weights of the Korea National Health and Nutrition Examination Survey (2019–2022) were applied to compute estimated population and prevalence to show nationally representative results.

^c^Computed from Rao-Scott Chi-Square test for weighted population to address multistage, complex, stratified probability-cluster sampling design of the Korea National Health and Nutrition Examination Survey (2019–2022) for categorical variables and survey regression for continuous variables.

^d^Calculated as the square root adjusted mean, dividing the total income of all household members by the square root of the number of household members.

^e^Lifetime non-drinker or consuming less than one drink per month in the past year.

^f^Consuming more than one drink per month in habitually, especially in the past year.

^g^Engaging in at least 150 minutes of moderate-intensity physical activity or 75 minutes of vigorous physical activity per week, or a combination of both.

^h^Defined as the percentage of respondents who needed and were being able to subsequently receive medical care (excluding dental) in the past 12 months.

^i^Assessed by asking respondents if they experienced limitations in daily and social activities due to any health problems.

^j^National health screening program provided by the National Health Insurance Service.

When influenza-vaccination status was examined according to household income and diabetes status (Table 2), vaccination rates ranged from 78.2% among participants with high income and no T2DM to 85.8% among those with low income and T2DM. After multivariable adjustment, individuals with both low income and T2DM had significantly lower odds of vaccination than those with high income and no T2DM (adjusted odds ratio [aOR], 0.75; 95% CI, 0.57–0.99). Participants with low income but without T2DM also tended to have lower vaccination odds (aOR, 0.82; 95% CI, 0.65–1.03), whereas high-income adults with T2DM did not differ significantly from the reference group (aOR, 1.12; 95% CI, 0.78–1.61).

**Table 2:** Combined association of household income status and diabetes with influenza vaccination in adults aged 65 years or older in the Korean National Health and Nutrition Examination Survey (2019-2022).

	High household income (≥Q3) and no T2DM	High household income (≥Q3) and T2DM	Low household income (≤Q2) and no T2DM	Low household income (≤Q2) and T2DM
**No. of participants, n (unweighted)**	998	383	2,910	1,234
**Estimated population, n (weighted)**	5,471,994	2,086,176	13,628,041	5,729,680
**Vaccination rate, n(%)**				
**Unweighted**	788 (79.0)	306 (79.9)	2,435 (83.7)	1,063 (86.1)
**Weighted**	4,277,918 (78.2)	1,624,150 (77.9)	1,1302,911 (82.9)	4,917,015 (85.8)
**Absolute differences, %**				
**Unweighted**	[Reference]	+0.9	+4.7	+7.1
**Weighted**	[Reference]	−0.3	+4.7	+7.6
**OR (95% CI)**				
**Model 1** ^**a**^	1 [Reference]	1.06 (0.75-1.50)	0.87 (0.69-1.09)	0.71 (0.54-0.93)^*^
**Model 2** ^**b**^	1 [Reference]	1.01 (0.71-1.45)	0.82 (0.65-1.03)	0.67 (0.51-0.88)^*^
**Model 3** ^**c**^	1 [Reference]	1.12 (0.78-1.61)	0.82 (0.65-1.03)	0.75 (0.57-0.99)^*^

NOTE: Sampling weights of the Korea National Health and Nutrition Examination Survey (2019–2022) were applied to compute estimated population and prevalence to obtain nationally representative results in multiple logistic regression analysis.

^a^Adjusted for age and sex (minimally-adjusted model) in the multivariable logistic regression model (weighted).

^b^Adjusted for age, sex, residential area, type of health insurance, marital status, alcohol consumption, cigarette smoking, physical activity, self-perceived health status, and health screening.

^c^Adjusted for comorbid conditions (hypertension, dyslipidemia, stroke, myocardial infarction, and angina pectoris), healthcare accessibility, and functional status in the multivariable logistic regression model (weighted) in addition to the variables included in Model 2 (weighted).

*P < 0.05.

Abbreviations/Acronyms: CI, confidence intervals; DM, diabetes mellitus; SES, socioeconomic status, OR, odds ratio

The combined association of education level and diabetes status with influenza vaccination is shown in Table 3. Vaccination coverage ranged from 77.9% among adults with higher education and no T2DM to 84.2% among those with lower education and T2DM. In adjusted analyses, the odds of vaccination were slightly lower—but not statistically significant—for participants with lower education and T2DM (aOR, 0.83; 95% CI, 0.61–1.15) and for those with lower education and no T2DM (aOR, 0.84; 95% CI, 0.63–1.13) compared with highly educated adults without T2DM.

**Table 3:** Combined association of education level and diabetes with influenza vaccination in adults aged 65 years or older in the Korean National Health and Nutrition Examination Survey (2019-2022).

	High education (≥college) and no T2DM	High education (≥college) and T2DM	Low education (≤high school) and no T2DM	Low education (≤high school) and T2DM
**No. of participants, n (unweighted)**	529	197	3,379	1,420
**Estimated population, n (weighted)**	2,846,706	1,084,238	16,253,329	6,731,618
**Vaccination rate, n(%)**				
**Unweighted**	415 (78.4)	160 (81.2)	2,808 (83.1)	1,209 (85.1)
**Weighted**	2,216,969 (77.9)	876,411 (80.8)	13,363,860 (82.2)	5,664,754 (84.2)
**Absolute differences, %**				
**Unweighted**	[Reference]	+2.8	+4.7	+6.7
**Weighted**	[Reference]	+2.9	+4.3	+6.3
**OR (95% CI)**				
**Model 1**	1 [Reference]	0.92 (0.58-1.47)	0.85 (0.64-1.13)	0.75 (0.56-1.01)
**Model 2**	1 [Reference]	0.88 (0.55-1.38)	0.83 (0.62-1.11)	0.73 (0.53-1.00)
**Model 3**	1 [Reference]	0.97 (0.62-1.53)	0.84 (0.63-1.13)	0.83 (0.61-1.15)

NOTE: Sampling weights of the Korea National Health and Nutrition Examination Survey (2019–2022) were applied to compute estimated population and prevalence to obtain nationally representative results in multiple logistic regression analysis.

^a^Adjusted for age and sex (minimally-adjusted model) in the multivariable logistic regression model (weighted).

^b^Adjusted for age, sex, residential area, type of health insurance, marital status, alcohol consumption, cigarette smoking, physical activity, self-perceived health status, and health screening.

^c^Adjusted for comorbid conditions (hypertension, dyslipidemia, stroke, myocardial infarction, and angina pectoris), healthcare accessibility, and functional status in the multivariable logistic regression model (weighted) in addition to the variables included in Model 2 (weighted).

*P < 0.05.

Abbreviations/Acronyms: CI, confidence intervals; DM, diabetes mellitus; SES, socioeconomic status, OR, odds ratio.

As shown in Table 4, vaccination coverage did not materially differ according to economic activity. Weighted rates ranged from 80.0% among economically active adults without T2DM to 84.8% among economically inactive adults with T2DM. Fully adjusted odds of vaccination were comparable across all categories; for example, the economically inactive with T2DM had an aOR of 0.91 (95% CI, 0.70–1.18) relative to the economically active without T2DM.

**Table 4:** Combined association of economic activity and diabetes with influenza vaccination in adults aged 65 years or older in the Korean National Health and Nutrition Examination Survey (2019-2022).

	Economically active and no T2DM	Economically active and T2DM	Economically inactive and no T2DM	Economically inactive and T2DM
**No. of participants, n (unweighted)**	1563	585	2345	1032
**Estimated population, n (weighted)**	7,359,206	2,779,245	11,740,829	5,036,612
**Vaccination rate, n(%)**				
**Unweighted**	1,266 (81.0)	490 (83.8)	1,957 (83.5)	879 (85.2)
**Weighted**	5,887,825 (80.0)	2,272,603 (81.8)	9,693,005 (82.6)	4268562 (84.8)
**Absolute differences, %**				
**Unweighted**	[Reference]	+2.8	+2.5	+4.2
**Weighted**	[Reference]	+1.8	+2.6	+4.8
**OR (95% CI)**				
**Model 1**	1 [Reference]	0.89 (0.66-1.21)	0.97 (0.79-1.18)	0.85 (0.66-1.09)
**Model 2**	1 [Reference]	0.89 (0.66-1.21)	0.92 (0.75-1.13)	0.80 (0.62-1.04)
**Model 3**	1 [Reference]	0.99 (0.73-1.35)	0.92 (0.75-1.14)	0.91 (0.70-1.18)

NOTE: Sampling weights of the Korea National Health and Nutrition Examination Survey (2019–2022) were applied to compute estimated population and prevalence to obtain nationally representative results in multiple logistic regression analysis.

^a^Adjusted for age and sex (minimally-adjusted model) in the multivariable logistic regression model (weighted).

^b^Adjusted for age, sex, residential area, type of health insurance, marital status, alcohol consumption, cigarette smoking, physical activity, self-perceived health status, and health screening.

^c^Adjusted for comorbid conditions (hypertension, dyslipidemia, stroke, myocardial infarction, and angina pectoris), healthcare accessibility, and functional status in the multivariable logistic regression model (weighted) in addition to the variables included in Model 2 (weighted)

*P < 0.05.

Abbreviations/Acronyms: CI, confidence intervals; DM, diabetes mellitus; SES, socioeconomic status, OR, odds ratio.

The period-stratified analysis comparing the pre–COVID-19 (2019) and COVID-19 (2020–2022) periods is summarized in Table 5. The overall pattern of associations between socioeconomic status, T2DM, and vaccination remained consistent across periods, and no significant interactions were observed for household income (*P* for interaction = 0.24), education (*P* = 0.51), or economic activity (*P* = 0.37). For instance, the aOR for low-income adults with T2DM compared with high-income adults without T2DM was 0.86 (95% CI, 0.46–1.61) in 2019 and 0.74 (95% CI, 0.54–1.01) in 2020–2022.

**Table 5:** Association between socioeconomic status, type 2 diabetes mellitus, and influenza vaccination among adults aged 65 years or older in the pre–COVID-19 (2019) and COVID-19 era (2020–2022) in the Korean National Health and Nutrition Examination Survey.

	Pre-COVID-19 era (2019)		COVID-19 era (2020–2022)		*P*-value for interaction
	Vaccination rate, n (%)	OR (95% CI)	Vaccination rate, n (%)	OR (95% CI)	
**Household income and T2DM**					
High household income (≥Q3) and no T2DM	85.3	1 [Reference]	76.4	1 [Reference]	0.239
High household income (≥Q3) and T2DM	80.2	1.63 (0.71-3.74)	77.3	1.04 (0.69-1.55)	
Low household income (≤Q2) and no T2DM	84.8	1.21 (0.76-1.92)	82.3	0.75 (0.57-0.97)	
Low household income (≤Q2) and T2DM	90.4	0.86 (0.46-1.61)	84.3	0.74 (0.54-1.01)	
**Education and T2DM**					
High education (≥college) and no T2DM	85.0	1 [Reference]	75.9	1 [Reference]	0.507
High education (≥college) and T2DM	83.4	1.46 (0.44-4.8)	80.2	0.91 (0.56-1.48)	
Low education (≤high school) and no T2DM	84.9	1.36 (0.74-2.49)	81.4	0.76 (0.54-1.05)	
Low education (≤high school) and T2DM	88.9	1.11 (0.55-2.22)	82.6	0.78 (0.55-1.12)	
**Economic activity and T2DM**					
Economically active and no T2DM	82.0	1 [Reference]	79.5	1 [Reference]	0.366
Economically active and T2DM	85.5	0.84 (0.44-1.59)	80.7	1.03 (0.72-1.48)	
Economically inactive and no T2DM	86.6	0.72 (0.47-1.12)	81.3	0.99 (0.78-1.25)	
Economically inactive and T2DM	89.8	0.67 (0.37-1.20)	83.2	0.99 (0.74-1.31)	

NOTE: Sampling weights of the Korea National Health and Nutrition Examination Survey (2019–2022) were applied to compute estimated population and prevalence to obtain nationally representative results in multiple logistic regression analysis.

Odds ratios and 95% confidence intervals shown above are adjusted for age, sex, residential area, type of health insurance, marital status, alcohol consumption, cigarette smoking, physical activity, self-perceived health status, and health screening, comorbid conditions (hypertension, dyslipidemia, stroke, myocardial infarction, and angina pectoris), healthcare accessibility, and functional status

Abbreviations/Acronyms: CI, confidence intervals; DM, diabetes mellitus; SES, socioeconomic status, OR, odds ratio

## 4. Discussion

In this nationally representative analysis of older adults in the Republic of Korea, we found a clear association between socioeconomic status (SES), type 2 diabetes mellitus (T2DM), and influenza vaccination uptake. Lower SES was consistently linked with reduced vaccination coverage, and this gap was more evident among individuals with T2DM. These findings illustrate how economic and clinical vulnerabilities can combine to limit participation in preventive health programs and identify an important area for policy and practice improvement.

The lower vaccination rates among people with limited income observed in this study agree with earlier work showing that financial strain can hinder access to preventive care, even within publicly supported health systems [3,27,28]. A comparable pattern was seen in analyses from the U.S. National Health and Nutrition Examination Survey, which showed that income-related gaps in vaccination coverage persist despite broad program availability [29]. In the Republic of Korea, influenza vaccination is provided without charge to adults aged 65 years and older through the National Immunization Program [30], however, indirect costs such as travel, time away from work, and the need for family support may still discourage participation among low-income groups. These observations suggest that improving vaccine uptake requires attention to practical barriers as well as direct costs.

Education level also showed a strong relationship with vaccination behavior. Participants with more years of schooling were more likely to be vaccinated, a finding consistent with previous evidence that education influences health literacy and the ability to navigate healthcare systems. A systematic review of nine studies on health literacy (HL) and vaccine hesitancy reported that HL affects vaccine acceptance in ways that vary by setting, age, and vaccine type [31]. Our results add to this evidence, indicating that educational advantage may translate into greater understanding of preventive benefits and confidence in vaccination. More longitudinal research using consistent measures of HL would help clarify these relationships.

Despite widespread awareness of vaccination benefits, many older adults with T2DM remain unvaccinated. A meta-analysis study showed that higher education levels were associated with greater influenza-vaccination coverage among adults with T2DM. Biological factors also play a role: immune responses to vaccination are often weaker in older adults because of aging-related immune decline and the inflammatory nature of diabetes, which together may limit vaccine effectiveness [2,32,33]. These findings underline the need for public-health messages that explain both the continuing value of vaccination and the potential limitations of immune response in older adults.

The lower vaccination rates among diabetic individuals from disadvantaged backgrounds are particularly concerning, given their increased risk of severe influenza and its complications. Limited health literacy, restricted healthcare access, and financial difficulties likely interact to reduce vaccination in these groups. Addressing these issues will require practical steps such as reducing out-of-pocket expenses, providing vaccination services in community clinics and local health centers, and ensuring that information is clear and easy to understand.

Several explanations may underlie the observed associations between SES, T2DM, and vaccination rates. Limited HL, often associated with lower educational attainment, may hinder awareness of vaccination importance or lead to misunderstandings about vaccine safety. Additionally, healthcare access challenges, which can be more pronounced in low SES populations, may act as significant barriers to vaccination uptake. These factors, combined with potential financial constraints, create a complex web of obstacles that must be addressed to improve vaccination rates in vulnerable populations. Our findings address the importance of public health strategies to improve influenza vaccination coverage among older adults, particularly those with T2DM and from lower SES backgrounds. Such strategies should include targeted education campaigns that use clear, accessible language and culturally sensitive messaging to address common concerns and misconceptions about vaccination. Efforts to reduce or eliminate out-of-pocket costs associated with vaccination, even when subsidies exist, could further encourage uptake among low-income individuals. Moreover, increasing the convenience of vaccination services through community-based clinics, mobile vaccination units, and partnerships with local pharmacies could help overcome logistical barriers to access.

This study has several strengths and limitations that should be considered when interpreting the findings. The use of nationally representative KNHANES data allowed for comprehensive analysis of influenza vaccination patterns across diverse socioeconomic and clinical groups among older adults in Korea. The large sample size and weighted analytic approach improved the generalizability of our results, while stratification by diabetes status and SES indicators enabled a more refined understanding of health inequalities in preventive care.

However, several limitations warrant discussion. First, influenza vaccination was assessed through self-reported responses, which may introduce recall bias or misclassification. Although previous validation studies suggest reasonable reliability of self-reported vaccination status in national health surveys, some degree of measurement error is inevitable. Second, the cross-sectional design limits causal inference, as the temporal sequence between exposure (SES and T2DM) and outcome (vaccination) cannot be definitively established. Longitudinal data would be needed to confirm directionality and persistence of these associations. Third, while extensive adjustments were made for demographic, behavioral, and health-related confounders, residual confounding by unmeasured variables such as physician recommendation, perceived vaccine efficacy, or regional differences in healthcare accessibility—cannot be ruled out. Fourth, although the study covered both pre–COVID-19 and pandemic periods, the rapid changes in healthcare utilization and vaccination behavior during the pandemic may have influenced overall vaccination patterns in ways that could not be fully captured by available data.

Despite these limitations, this analysis provides robust population-based evidence linking lower SES and diabetes with reduced influenza vaccination coverage among older adults. The findings are consistent with prior studies demonstrating that social and clinical vulnerabilities compound disparities in preventive healthcare. Economic hardship, limited health literacy, and restricted access to services likely act together to reduce vaccination uptake in these groups. These results underscore the importance of policy measures that go beyond cost subsidies to address the practical and informational barriers faced by vulnerable populations.

Future studies should leverage longitudinal or claims-based datasets to examine how structural and behavioral factors evolve over time and to assess the effectiveness of interventions designed to increase vaccination among high-risk groups. Integration of health literacy assessment and regional health-service metrics may also provide valuable insight into mechanisms underlying inequities in vaccine uptake.

In conclusion, lower socioeconomic position and diabetes were jointly associated with reduced influenza vaccination among older adults in Korea. These results highlight the continued influence of social disadvantage on preventive health behavior despite the existence of a universal immunization program. Reducing these disparities requires strengthening community-based vaccination initiatives, improving communication about vaccine benefits, and ensuring that preventive programs reach those at greatest risk. Addressing these inequities is essential not only for mitigating influenza-related morbidity and mortality but also for advancing health equity in an aging society.
